# Supplementary material for: Asynchronous magnetic resonance elastography: Shear wave speed reconstruction using noise correlation of incoherent waves
Source: Magn Reson Med. 2022 Oct 27;89(3):990–1001. doi: 10.1002/mrm.29502 (PMC9792433; doi:10.1002/mrm.29502)
Supplement: Supplementary file 1 — DATA S1 MATLAB code used to generate the numerical simulations of Figure 1 [file MRM-89-990-s002.zip › k-Wave/helpfiles/example_sd_focussed_detector_3D.html]

Focussed Detector In 3D Example (k-Wave)


# Focussed Detector In 3D Example

This example shows how k-Wave can be used to model the output of a focussed bowl detector where the directionality arises from spatially averaging across the detector surface. It builds on the Focussed Detector In 2D example.

- Open the file in the MATLAB Editor
- Run the file in MATLAB

## Contents

- Defining the sensor geometry
- Defining on and off-axis point sources
- Running the simulations

## Defining the sensor geometry

For large single element detectors, the directionality arises because the output is spatially averaged across the pressure field on the transducer surface. Here this effect is simulated using a focussed bowl detector (defined using a binary sensor mask) created with the help of `makeBowl`.

```
% create a concave sensor
sphere_offset = 10;                             % [grid points]
diameter = Nx/2 + 1;                            % [grid points]
radius = Nx/2;                                  % [grid points]
bowl_pos = [1 + sphere_offset, Ny/2, Nz/2];     % [grid points]
focus_pos = [Nx/2, Ny/2, Nz/2];                 % [grid points]
sensor.mask = makeBowl([Nx, Ny, Nz], bowl_pos, radius, diameter, focus_pos);
```

## Defining on and off-axis point sources

To see the focussing effect of the bowl detector, two points sources are defined, one on-axis and one off-axis.

```
% place the first point source near the focus of the detector
source1 = zeros(Nx, Ny, Nz);
source1(1 + sphere_offset + radius, Ny/2, Nz/2) = 1;

% place the second point source off axis
source2 = zeros(Nx, Ny, Nz);
source2(1 + sphere_offset + radius, Ny/2 + 6, Nz/2 + 6) = 1;
```


## Running the simulations

Following the simulations, the recorded time series (one per detector grid point) are averaged to model the output of a single element focussed sensor. The resulting time series for both on and off axis source positions are shown below. The detector clearly produces a larger output for the source nearer its focus.

```
% run the first simulation
source.p_mask = source1;
input_args = {'PMLSize', 10, 'DataCast', 'single', 'PlotSim', false};
sensor_data1 = kspaceFirstOrder3D(kgrid, medium, source, sensor, input_args{:});

% average the data recorded at each grid point to simulate the measured
% signal from a single element focussed detector
sensor_data1 = sum(sensor_data1, 1);

% run the second simulation
source.p_mask = source2;
sensor_data2 = kspaceFirstOrder3D(kgrid, medium, source, sensor, input_args{:});

% average the data recorded at each grid point to simulate the measured
% signal from a single element focussed detector
sensor_data2 = sum(sensor_data2, 1);
```
